# Blood‐brain barrier integrity and vessel morphology in adults with Down syndrome and Alzheimer's neuropathology

**DOI:** 10.1002/alz70855_106199

**Published:** 2025-12-24

**Authors:** Lisi Flores Aguilar, Jesse R. Pascual, Halyma Nguyen, Annie Du, Natalie C. Edwards, Patrick J. Lao, Jae Kyu Ryu, Katerina Akassoglou, Donna M. Wilcock, Julia K. Kofler, Milos D. Ikonomovic, Florence Lai, Adam Brickman, Elizabeth Head, Alzheimer's Biomarker Consortium — Down Syndrome (ABC‐DS)

**Affiliations:** ^1^ University of California, Irvine, Irvine, CA, USA; ^2^ Columbia University, New York, NY, USA; ^3^ Gladstone Institutes, UCSF, San Francisco, CA, USA; ^4^ Indiana University School of Medicine, Department of Neurology, Indianapolis, IN, USA; ^5^ University of Pittsburgh, Pittsburgh, PA, USA; ^6^ Harvard/Massachusetts General Hospital, Boston, MA, USA

## Abstract

**Background:**

In the coming year, adults with Down syndrome (DS) will be included in Alzheimer's disease (AD) clinical trials involving anti‐amyloid immunotherapies. Such therapies have been associated with adverse cerebrovascular events such as amyloid‐related imaging abnormalities (ARIA). Moreover, cerebral amyloid angiopathy (CAA) is associated with an increased risk of ARIA. By the age of 40 years, people with DS exhibit AD neuropathology (DSAD) and cerebrovascular disease, including severe CAA, suggesting that individuals with DS may be at increased risk of ARIA. Thus, to better understand their cerebrovascular profile, our main objective is to characterize cerebrovascular pathology in individuals with DS, including blood‐brain barrier (BBB) integrity and vascular morphology.

**Methods:**

Free‐floating immunohistochemistry was used to label the basement membrane (BM), blood vessels, pericytes, and fibrin parenchymal deposition in the occipital cortex of adults with DSAD (*n* = 12) and age‐matched neurotypical controls (*n* = 12). We measured basement membrane coverage, vessel length, and density, and the number of pericytes and string vessels (also known as collapsed capillaries or connective tissue strands with no endothelial cells) in lamina III‐IV. Fibrin deposition in the brain parenchyma was visually scored using whole brain sections. Two‐tailed unpaired t‐tests, Mann‐Whitney, and Fisher's exact test were used accordingly. We are analyzing the same measures in a second and larger autopsy cohort from young (1‐33 years old, *n* = 14) and old individuals with DS (42‐70 years old, *n* = 84), late‐onset AD (75‐90 years old, *n* = 33), and age‐matched neurotypical controls (2‐90 years old, *n* = 72).

**Results:**

Compared to neurotypical controls, adults with DSAD displayed increased BM coverage [t(18)=2.145, *p* = 0.045], vessel density [t(18)=5.277, *p* <0.0001] and length [t(18)=3.43, *p* = 0.003]. We also found an increased number of string vessels (U=13, *p* = 0.0009) and pericytes [t(18)=3, *p* = 0.005]. Pericyte per vessel density did not differ between groups [t(18)=1.813, *p* = 0.086]. Finally, we found abundant fibrin deposition in DSAD brains compared to controls (3‐fold increase, Fisher's exact test, two‐tailed, *p* = 0.0002).

**Conclusion:**

Adults with DS at late stages of AD neuropathology display vascular changes suggestive of vascular remodeling processes and BBB breakdown. The administration of anti‐amyloid beta immunotherapies in this population must be carefully evaluated.

Funding: NIH‐U19AG068054, RF1AG079519, P30AG066519, 23AARFD‐1022715.

